# Hypoxia is associated with a lower expression of genes involved in lipogenesis in visceral adipose tissue

**DOI:** 10.1186/s12967-015-0732-5

**Published:** 2015-11-30

**Authors:** Eduardo García-Fuentes, Concepción Santiago-Fernández, Carolina Gutiérrez-Repiso, María D. Mayas, Wilfredo Oliva-Olivera, Leticia Coín-Aragüez, Juan Alcaide, Luis Ocaña-Wilhelmi, Joan Vendrell, Francisco J. Tinahones, Lourdes Garrido-Sánchez

**Affiliations:** Department of Endocrinology and Nutrition, Institute of Biomedical Research of Malaga (IBIMA), Regional University Hospital, Malaga, Spain; CIBEROBN, Institute of Health Carlos III, Malaga, Spain; Department of Endocrinology and Nutrition, Institute of Biomedical Research of Malaga (IBIMA), Virgen de la Victoria Clinical University Hospital, Malaga, Spain; Department of Physiology, University of Jaen, Jaén, Spain; Department of Surgery, Institute of Biomedical Research of Malaga (IBIMA), Virgen de la Victoria Clinical University Hospital, Malaga, Spain; CIBERDEM, Institute of Health Carlos III, Tarragona, Spain; Department of Endocrinology and Nutrition, Joan XXIII University Hospital, Pere Virgili Institute, Rovira i Virgili University, Tarragona, Spain; Laboratorio de Investigación, Hospital Civil, Plaza del Hospital Civil s/n, 29009 Málaga, Spain

**Keywords:** HIF-1α, Hypoxia, Adipose tissue, Insulin resistance, Morbid obesity

## Abstract

**Background:**

A key role for HIF-1α in the promotion and maintenance of dietary obesity has been proposed. We analyzed the association between hypoxia and de novo lipogenesis in human adipose tissue.

**Methods:**

We studied HIF-1α mRNA and protein expression in fasting status in visceral adipose tissue (VAT) from non-obese and morbidly obese subjects, and in VAT from wild-type and ob/ob C57BL6J mice in both fasting and feeding status. We also analyzed the effect of hypoxia on the VAT mRNA expression of genes involved in lipogenesis.

**Results:**

HIF-1α was increased in VAT from morbidly obese subjects. In fasting status, C57BL6J ob/ob mice had a higher VAT HIF-1α mRNA expression than C57BL6J wild-type mice. In feeding status, VAT HIF-1α mRNA expression significantly increased in C57BL6J wild-type, but not in C57BL6J ob/ob mice. In humans, HIF-1α mRNA expression correlated positively with body mass index and insulin resistance. VAT HIF-1α mRNA expression correlated negatively with ACC1, PDHB and SIRT3 mRNA expression, and positively with PPAR-γ. VAT explants incubated in hypoxia showed reduced SIRT3 and increased PPAR-γ, SREBP-1c, ACLY, ACC1 and FASN mRNA expression.

**Conclusions:**

Morbidly obese subjects have a higher level of VAT HIF-1α. Postprandial status is associated with an increase in HIF-1α mRNA expression in C57BL6J wild-type mice. Hypoxia alters the mRNA expression of genes involved in de novo lipogenesis in human VAT.

## Background

Hypoxia has been involved in the pathogenesis of several human diseases [1]. Hypoxia mainly mediates its effect through the activation of hypoxia-inducible factor (HIF), a transcription factor composed of two subunits, HIF-1α and β. HIF-1β is constitutively expressed and not regulated directly by O_2_ [2]. There are at least three α-subunits of HIF-1, HIF-1α, HIF-2α and HIF-3α, and the combination of any of these with HIF-1β forms the functional transcription factor. HIF-1α appears to be the most important. In hypoxia, there is no degradation of HIF-1, and stabilized HIF-1 protein translocates to the nucleus and modifies the transcription of different genes [3].

The correct function of adipose tissue is of vital importance to avoid different obesity-associated disorders [4, 5]. Moreover, a previous study has shown that the status of different pathways involved in fatty acid metabolism may be involved in the improvement of morbidly obese subjects after bariatric surgery [6]. Hypoxia affects a number of biological functions, such as angiogenesis, cell proliferation, apoptosis, inflammation and insulin resistance [7, 8]. Different studies suggest that adipose tissue is poorly oxygenated in obese humans and mice, resulting in the induction of HIF-1α [5, 9]. In most studies in obese people, fasting subcutaneous adipose tissue blood flow (ATBF) is reduced compared with lean people [10]. Also, ATBF increases rapidly after a meal [11], but this response to meal ingestion is diminished, or lost entirely, in obese people [10]. However, little information is available on its postprandial effect on human visceral adipose tissue (VAT) hypoxia. This issue could be important since humans are in a feeding status most of the day. In addition, there is a close association between VAT oxygenation status and the development of obesity [12]. The postprandial ATBF response has also been shown to be related to insulin sensitivity, independent of body mass index (BMI) [13]. Other studies also suggest that adipose tissue plays an important role in the development of insulin resistance [14]. A direct effect of hypoxia in inducing insulin resistance in 3T3-L1 adipocytes has been documented [15].

Some studies have related hypoxia to the expression of genes associated with lipid metabolism, such as peroxisome proliferator-activated receptor-gamma (PPAR-γ) and sterol regulatory element-binding proteins-1c (SREBP-1c) [16]. A key role for HIF-1α has been proposed in the promotion and maintenance of dietary obesity, at least in part, by suppressing adipocyte lipid catabolism [17]. However, most of these studies were conducted in rodents and in tissues different to adipose tissue. There is very little reported on the influence of hypoxia on de novo lipogenesis in human VAT. Different genes involved in this pathway could be modified in the hypoxia condition, such as ATP citrate lyase (ACLY) (catalyzes the synthesis of cytosolic acetyl-CoA from citrate) [18], acyl-CoA synthetase short-chain family member 2 (ACSS2) (catalyzes the activation of cytosolic acetate to acetyl-CoA) [19], pyruvate dehydrogenase (lipoamide) beta (PDHB) (converts pyruvate into acetyl-CoA in mitochondria) [20], acetyl-CoA carboxylase-α (ACC1) (catalyzes the carboxylation of acetyl-CoA to malonyl-CoA) [21] and fatty acid synthase (FASN) (catalyzes the condensation of acetyl-CoA and malonyl-CoA to produce palmitic acid) [22]. Also, sirtuin-3 (SIRT3) has been proposed as a major control point for obesity-related metabolic diseases and it can regulate the activity of some of these genes [23].

Since hypoxia could be related to adipocyte lipid synthesis, we studied the relationship between HIF-1α, a marker of hypoxia, and the mRNA expression of genes involved in lipogenesis in human VAT. First, in the present study we analyzed the association between VAT HIF-1α and several obesity-related variables and the mRNA expression of genes involved in lipogenesis. Since in previous studies we have demonstrated that insulin resistance was associated with the expression of genes involved in lipid metabolism and with other proteins in adipose tissue [24–28], we wanted to determine whether insulin resistance is also associated with HIF-1α. Second, we tested in vitro the effect of hypoxia on the mRNA expression of genes involved in lipogenesis. We have performed this study in VAT since there is a close association between the VAT oxygenation status and the development of obesity [12]. Also, a recent study has shown that although delivery of O_2_ to the obese subcutaneous adipose tissue (SAT) is reduced, no evidence of a metabolic signature is found to support the notion of obesity-related SAT hypoxia in the fasting and postprandial states [29]. Third, as humans are in a feeding status most of the day [30], we tested whether this state produces a change in VAT HIF-1α expression. There is little information on the effect of the postprandial state on HIF-1α expression in human VAT. However, given the impossibility of access to human VAT in the postprandial state, we used C57BL6J mice to test whether this state produces a change in the HIF-1α mRNA expression in both fasting and feeding status.

## Methods

### Subjects

We evaluated 30 morbidly obese subjects, 15 with low insulin resistance [homeostasis model assessment of insulin resistance index (HOMA-IR) <4.7] and 15 with high insulin resistance (HOMA-IR >8) [24]. All the morbidly obese subjects underwent biliopancreatic diversion of Scopinaro. We also studied 15 non-obese subjects who underwent laparoscopic surgery for cholelithiasis. Subjects were excluded if they were receiving insulin or hypoglycemic agents, had cardiovascular disease, arthritis, acute inflammatory disease, infectious disease, or were receiving drugs that could alter the lipid profile or the metabolic parameters at the time of inclusion in the study. All subjects were of Caucasian origin. All participants gave their written informed consent and the study was reviewed and approved by the Ethics and Research Committee of Virgen de la Victoria Clinical University Hospital, Malaga, Spain.

### Laboratory measurements

Blood samples were collected after a 12-h fast. The serum was separated and immediately frozen at −80 °C. Serum biochemical parameters were measured in duplicate. Serum glucose, cholesterol, triglycerides and free fatty acids (FFA) were measured by standard enzymatic methods (Randox Laboratories Ltd., Antrium, UK). Total adiponectin levels were measured by enzyme immunoassay (ELISA) kits (DRG Diagnostics, Marburg, Germany). Leptin levels were measured by ELISA kit from Mediagnost (Reutlingen, Germany). The insulin was analyzed by an immunoradiometric assay (BioSource International, Camarillo, CA). The HOMA-IR was calculated from fasting insulin and glucose: HOMA-IR = fasting insulin (μIU/mL) × fasting glucose (mol/L)/22.5.

### Human adipose tissue samples

All reagents were from Sigma-Aldrich (Sigma-Aldrich, St. Louis, MO) unless otherwise indicated. VAT samples were obtained during bariatric surgery in the morbidly obese subjects (n = 30) and during laparoscopic surgery in the non-obese subjects (n = 15) [6, 24, 25]. Biopsy samples were immediately washed in physiological saline and frozen in liquid nitrogen after being obtained from the subject, and then stored at −80 °C until analysis. Another VAT sample from the non-obese subjects was placed in phosphate buffered saline (PBS) supplemented with 5 % bovine serum albumin (BSA) to perform adipose tissue explant cultures.

### Human adipose tissue culture

VAT explants from non-obese subjects were prepared by cutting samples into 5 mg portions, which were subsequently incubated for 30 min in PBS + 5 % BSA (3 ml/g) [26]. After 30 s of centrifugation (400*g*), samples were incubated in M199 medium (Life Technologies, Grand Island, NY) supplemented with 10 % fetal bovine serum (FBS), 100 U/mL penicillin and streptomycin. VAT explants were incubated for 24 h at 37 °C in 95 % air and 5 % CO_2_ (normoxic conditions) or placed in a hypoxic chamber (Billups-Rothenberg, Dell Mar, CA) at 37 °C and 1 % O_2_, 5 % CO_2_, and 94 % N_2_ (hypoxic conditions) (n = 5). Following these treatments, VAT explants were immediately collected and frozen in liquid nitrogen, and then stored at −80 °C until analysis.

### Animal study

Thirty-four C57BL6J wild-type mice and the same number of C57BL6J ob/ob mice (body weight 18.7 ± 1.8 and 37.5 ± 2.5 g, respectively) were used. The animals were obtained from the animal care facility of Malaga University and were housed under constant temperature conditions (20–25 °C) and day length 12 h. C57BL6J wild-type mice and C57BL6J ob/ob mice were randomly divided into two groups: one group of mice was fed ad libitum and the other group was withheld food for 24 h before being sacrificed. All groups were allowed access to tap water ad libitum. The experimental procedures for animal use and care were in accordance with the European Community Council Directive 86/609/EEC. Protocols were approved by the Bioethical Committee of Virgen de la Victoria Clinical University Hospital, Malaga, Spain. Animals were sacrificed by decapitation and VAT biopsies were immediately frozen in liquid nitrogen and then stored at −80 °C for gene expression analysis.

### RNA extraction and real-time quantitative PCR

Frozen VAT from human, mice and explant cultures was introduced in Trizol (QIAGEN Science, Hilden, Germany), thawed and immediately homogenized with an Ultra-Turrax 8 (Ika, Staufen, Germany). Total RNA was obtained by RNeasy lipid tissue midi kit (QIAGEN Science, Hilden, Germany) as previously described [6, 27]. Total RNA was reverse transcribed to cDNA by using a high-capacity cDNA reverse transcription kit with RNase inhibitor (Applied Biosystems, Foster City, CA). The cDNA was used for quantitative real-time PCR in an ABI 7500 Fast Real-Time PCR System (Applied Biosystems, Foster City, CA). Reactions were carried out in duplicate for all genes using specific TaqMan^®^ Gene Expression Assays (Applied Biosystems, Foster City, CA): mouse HIF-1α (Mm00468869_m1, RefSeq. NM_010431.2), and human PPAR-γ (Hs01115510_m1, RefSeq. NM_015869.4), SREBP-1c (Hs00967385_g1, RefSeq. NM_001018067.1, NM_001018068.1, NM_001018069.1, NM_015640.3), HIF-1α (Hs00153153_m1, RefSeq. NM_001243084.1, NM_001530.3, NM_181054.2), PDHB (Hs00168650_m1, RefSeq. NM_000925.3, NM_001173468.1, NM_033384.1), SIRT3 (Hs00202030_m1, RefSeq. NM_001017524.2, NM_012239.5), ACLY (Hs00982738_m1, RefSeq. NM_001096.2, NM_198830.1), ACC1 (Hs00167385_m1, RefSeq. NM_198834.1, NM_198836.1, NM_198837.1, NM_198838.1, NM_198838.1), ACSS2 (Hs00218766_m1, RefSeq. NM_001076552.2, NM_001242393.1, NM_018677.3) and FASN (Hs00188012_m1, RefSeq. NM_004104.4). The cycle threshold (Ct) value for each sample was normalized with the expression of cyclophilin A (*PPIA)* (4326316E, RefSeq. NM_021130.3). SDS software 2.3 and RQ Manager 1.2 (Applied Biosystems, Foster City, CA) were used to analyze the results with the comparative Ct method (2^−ΔΔCt^). All data were expressed as an n-fold difference relative to the calibrator (a mixture of the SAT and VAT tissues was used as the calibrator sample).

### Western blot

Frozen VAT from humans and mice was thawed and immediately homogenized in RIPA buffer with Protease Inhibitor Cocktail (Sigma-Aldrich, St. Louis, MO) and centrifuged (13,000 rpm, 10 min, 4 °C) [28]. The concentration of proteins in the supernatant was determined according to the bicinchoninic acid method (Thermo Fisher Scientific Inc. Rockford, IL). 20 μg of protein was submitted to 10 % sodium dodecyl sulfate polyacrylamide gel electrophoresis, transferred to polyvinylidene difluoride membrane at 15 V for 1 h, and blocked in Protein-Free Tween 20 Blocking Buffer (Thermo Fisher Scientific Inc., Rockford, IL). After washing with PBS + 0.05 % Tween 20, membranes were incubated with a rabbit polyclonal antibody anti-HIF-1α (Santa Cruz Biotechnology, Santa Cruz, CA) or anti-β-actin (Sigma-Aldrich, St. Louis, MO) at a dilution of 1:800 or 1:1000, respectively, for 1 h at room temperature. Membranes were washed and incubated with horseradish peroxidase-conjugated secondary antibody (Promega, Madison, WI, USA) at a dilution of 1:1000 or 1:5000, respectively, for 1 h at room temperature. The proteins were visualized with SuperSignal^®^ West Pico Chemiluminescent Substrate (Pierce Biotechnology) and quantified by an Auto-Chemi System (UVP, Upland, CA, USA) and the image acquisition analysis software Labworks 4.6 (UVP). The results were expressed as HIF-1α/β-actin ratio.

### Enzymatic assays in human adipose tissue

All reagents were from Sigma-Aldrich (Sigma-Aldrich, St. Louis, MO) unless otherwise specified. Fresh biopsies of VAT from morbidly obese (n = 5) and non-obese subjects (n = 5), and VAT explant cultures from non-obese subjects (in normoxic and hypoxic conditions) (n = 3) were immediately washed with phosphate-buffered saline and homogenized in RIPA buffer on ice. The cell lysates were sonicated briefly followed by centrifugation (16,000*g*, 4 °C) for 15 min to remove insoluble materials and fat. The supernatant was used to analyze the PDH, ACLY and ACSS enzymatic activities.

PDH activity was determined using a protocol as previously described [31]. Briefly, 4 μl of supernatant of cell lysate was mixed with a buffer containing 87 mM triethanolamine/HCl pH 7.8, 2.0 mM MgCl_2_, 0.2 mM thiamin diphosphate, 5.0 mM pyruvate and 0.2 mM 2,6-dichlorophenolindophenol in a final volume of 0.2 ml. The absorption change for 5 min was pursued at 600 nm and 30 °C. Data are expressed as U/mg adipose tissue.

ACSS activity was determined using a protocol as previously described [32]. Briefly, 40 μl of supernatant of cell lysate was mixed with 140 μl of buffer containing 70 μl of 100 mM Tris–HCl buffer (pH 7.8), 10 μl of 50 mM l-malate, 10 μl of 20 mM ATP, 10 μl of 50 mM MgCl_2_, 10 μl of 2 mM Coenzyme A trilithium salt, 10 μl of 60 mM NAD^+^, 10 μl of 50 U/ml malate dehydrogenase from bovine heart and 10 μl of 25 U/ml citrate synthase from porcine heart. The reaction was started by adding of 20 μl of 1 M sodium acetate, incubated at 37 °C and the absorbance monitored at 340 nm for 10 min. Data are expressed as U/mg adipose tissue.

ACLY activity was determined using a protocol as previously described [33]. Briefly, 10 μl of supernatant of cell lysate was mixed with 290 μl of a buffer containing 96 mM triethanolamine, 0.5 mM zinc chloride, 0.23 mM β-NADH, 0.67 mM sodium citrate, 100 units l-lactic dehydrogenase, 50 units malic dehydrogenase and 15 mM ammonium sulfate, pH 7.6. The reaction was monitored at 340 nm for 5 min at room temperature. Data are expressed as U/mg adipose tissue.

### Malonyl-CoA in human adipose tissue

Malonyl-CoA concentration was analyzed by a commercial kit (BlueGene Biotech, Shanghai, China) in fresh biopsies of VAT from morbidly obese (n = 5) and non-obese subjects (n = 5), and in VAT explant cultures from non-obese subjects (in normoxic and hypoxic conditions) (n = 3) according to manufacturer’s instructions.

### Statistical analysis

The statistical analysis was done with SPSS (Version 11.5 for Windows; SPSS, Chicago, IL). Differences between the two groups were compared by the Student *t* test. Comparison between the results of the different groups was done with a univariate general linear model, adjusted for sex, and the post hoc analysis was done with the Bonferroni method. The Pearson correlation coefficients were calculated to estimate the correlations between variables. Multiple linear regressions were used to determine the association between HIF-1α mRNA expression and other variables. Values were considered to be statistically significant when *P* ≤ 0.05. The results are given as the mean ± SD, and as the mean ± SEM in figures.

## Results

### HIF-1α was increased in morbidly obese subjects

**Fig. 1:**
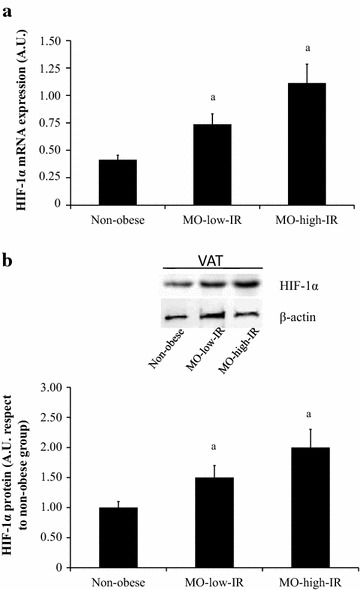
HIF-1α expression in visceral adipose tissue (VAT) in non-obese and morbidly obese subjects with low (MO-low-IR) and high insulin resistance (MO-high-IR) in fasting condition. **a** HIF-1α mRNA and **b** representative immunoblot from non-obese and morbidly obese subjects (n = 4 per group). The results are given as the mean ± SEM. ^a^P < 0.05 significant differences respect to the non-obese group. *A.U.* arbitrary units

Table 1 summarizes the characteristics of the different groups of subjects. HIF-1α mRNA expression in VAT was increased in the morbidly obese subjects (Fig. 1a). These results were confirmed by western blot analysis (n = 4 per group) (Fig. 1b).

**Table 1:** Anthropometric and biochemical variables in the non-obese and morbidly obese subjects classified according to their insulin resistance levels

	Non-obese subjects (N = 15)	MO-low-IR (N = 15)	MO-high-IR (N = 15)
Sex (male/female)	4/11	5/10	5/10
Age (years)	40.3 ± 11.8	40.6 ± 11.4	37.2 ± 9.4
Weight (kg)	63.1 ± 8.5	153.6 ± 29.5^a^	158.7 ± 23.0^a^
BMI (kg/m^2^)	22.5 ± 1.7	55.6 ± 7.2^a^	57.6 ± 4.8^a^
Waist (cm)	80.0 ± 8.7	140.8 ± 20.3^a^	147.5 ± 19.6^a^
Hip (cm)	91.3 ± 7.7	160.1 ± 15.8^a^	158.1 ± 13.8^a^
Glucose (mg/dl)	83.1 ± 10.7	91.1 ± 10.5^a^	107.9 ± 15.8^a^*
Cholesterol (mg/dl)	192.0 ± 42.6	201.4 ± 40.4	193.5 ± 31.3
Triglycerides (mg/dl)	89.6 ± 44.9	114.7 ± 55.4	163.7 ± 77.8^a^*
Free fatty acids (mmol/L)	0.305 ± 0.030	0.585 ± 0.066^a^	0.584 ± 0.042^a^
Insulin (μIU/ml)	5.9 ± 3.6	12.6 ± 4.1^a^	39.6 ± 10.4^a^*
HOMA-IR	1.2 ± 0.72	2.8 ± 0.81^a^	10.6 ± 2.4^a^*
Adiponectin (μg/mL)	29.8 ± 18.5	10.9 ± 4.0^a^	8.0 ± 4.7^a^
Leptin (ng/mL)	8.2 ± 8.03	77.6 ± 47.8^a^	59.8 ± 21.5^a^

The results are given as the mean ± standard deviation

*MO-low-IR* morbidly obese subjects with low insulin resistance, *MO-high-IR* morbidly obese subjects with high insulin resistance, *BMI* body mass index, *HOMA-IR* homeostasis model assessment of insulin resistance index

^a^P < 0.05 significant differences with respect to the non-obese group

* P < 0.05 significant differences between morbidly obese patients with low and high insulin resistance. All significant differences are adjusted for sex

### HIF-1α was associated with biochemical and anthropometric variables

HIF-1α mRNA expression in VAT correlated positively with different anthropometric variables, such as weight (r = 0.676, p < 0.001), BMI (r = 0.514, p = 0.001), waist (r = 0.602, p < 0.001) and hip circumferences (r = 0.470, p = 0.004), insulin (r = 0.470, p = 0.004) and HOMA-IR (r = 0.449, p = 0.008). HIF-1α mRNA expression in VAT correlated negatively with adiponectin (r = −0.418, p = 0.019). The variable which was associated with VAT HIF-1α mRNA expression in a multiple linear regression model was the HOMA-IR (β = 2.022, p = 0.047) (R^2^ = 0.242), after adjusting for sex, age, BMI, waist and hip circumferences, insulin and adiponectin.

### HIF-1α expression was associated with the expression of genes involved in de novo lipogenesis in human adipose tissue

Table 2 shows the expression of different genes involved in de novo lipogenesis in human VAT (PPAR-γ, SREBP-1c, ACLY, ACSS2, PDHB, ACC1, FASN and SIRT3). Morbidly obese subjects had a lower mRNA expression of SREBP-1c, ACLY, ACSS2, PDHB, ACC1 and FASN than non-obese subjects. The measure of the enzymatic activity confirmed the results of mRNA expression of ACLY (non-obese: 0.033 ± 0.002 vs. morbidly obese subjects: 0.021 ± 0.005 U/mg VAT, p = 0.026), ACCS (non-obese: 0.025 ± 0.017 vs. morbidly obese subjects: 0.007 ± 0.003 U/mg VAT, p = 0.016) and PDH (non-obese: 4.3 × 10^−8^ ± 0.20 × 10^−8^ vs. morbidly obese subjects: 1.1 × 10^−8^ ± 0.3 × 10^−8^ U/mg VAT, p = 0.048). ACC1 mRNA expression was confirmed by the analysis of malonyl-CoA concentration in VAT (non-obese: 0.126 ± 0.055 vs. morbidly obese subjects: 0.074 ± 0.025 ng/mg VAT, p = 0.048).

**Table 2:** mRNA expression of different genes involved in de novo lipogenesis in human visceral adipose tissue

	Non-obese subjects (N = 15)	MO-low-IR subjects (N = 15)	MO-high-IR subjects (N = 15)
SIRT3	0.029 ± 0.014	0.021 ± 0.015	0.009 ± 0.006^a^*
PDHB	0.460 ± 0.170	0.244 ± 0.066^a^	0.248 ± 0.081^a^
ACC1	1.87 ± 0.43	1.15 ± 0.52^a^	0.91 ± 0.59^a^
ACSS2	1.14 ± 0.29	0.78 ± 0.42^a^	0.80 ± 0.39^a^
ACLY	1.01 ± 0.79	0.42 ± 0.08^a^	0.31 ± 0.06^a^
PPARγ	1.12 ± 0.65	2.72 ± 1.89^a^	2.51 ± 0.87^a^
SREBP-1c	0.271 ± 0.172	0.131 ± 0.072^a^	0.116 ± 0.067^a^
FASN	0.285 ± 0.088	0.148 ± 0.040^a^	0.124 ± 0.020^a^

*MO-low-IR* morbidly obese subjects with low insulin resistance, *MO-high-IR* morbidly obese subjects with high insulin resistance

^a^P < 0.05 significant differences with respect to the non-obese group

* P < 0.05 significant differences between morbidly obese patients with low and high insulin resistance. All significant differences are adjusted for sex

We found a significant correlation between HIF-1α mRNA expression and ACC1 (r = −0.417, p = 0.009), SIRT3 (r = −0.346, p = 0.039), PDHB (r = −0.585, p = 0.037) and PPAR-γ mRNA expression (r = 0.395, p = 0.014).

### Hypoxia altered de novo lipogenic gene expression

**Fig. 2:**
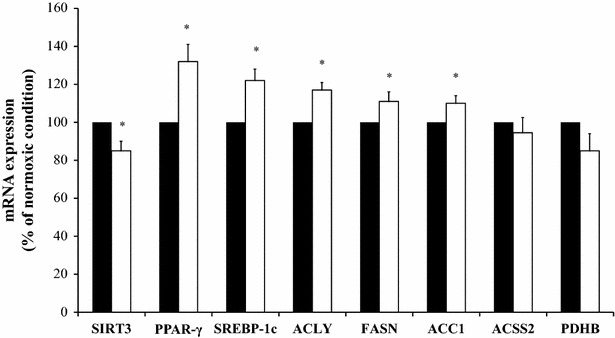
mRNA expression of SIRT3, PPAR-γ, SREBP-1c, ACLY, FASN, ACC1, ACSS2 and PDHB in visceral adipose tissue explants culture incubated for 24 h at 37 °C in normoxic conditions (*filled square*) or placed in a hypoxic chamber for 24 h at 37 °C in hypoxic conditions (*open square*) (n = 5 per group). Results are shown as a percentage of the normoxic condition. The results are given as the mean ± SEM. *P < 0.05 significant differences between normoxic and hypoxic condition

Hypoxia produced a significant reduction in the mRNA expression of SIRT3 (p = 0.033) (Fig. 2). However, hypoxia produced a significant increase in ACC1 (p = 0.011), ACLY (p = 0.020), PPAR-γ (p = 0.017), FASN (p = 0.011) and SREBP-1c mRNA expression (p = 0.034). The measure of the enzymatic activity confirmed the results of mRNA expression of ACLY (hypoxia: 0.064 ± 0.005 vs. normoxia: 0.029 ± 0.003 U/mg VAT, p = 0.002), ACCS (hypoxia: 0.0081 ± 0.0007 vs. normoxia: 0.0071 ± 0.0008 U/mg VAT, p = 0.186) and PDH (hypoxia: 1.4 × 10^−8^ ± 0.50 × 10^−8^ vs. normoxia: 1.6 × 10^−8^±1.0 × 10^−8^ U/mg VAT, p = 0.480). The ACC1 mRNA expression was confirmed by the analysis of malonyl-CoA concentration in VAT (hypoxia: 0.127 ± 0.010 vs. normoxia: 0.085 ± 0.003 ng/mg VAT, p = 0.012).

### Feeding status was associated with an increase of HIF-1α in visceral adipose tissue from C57BL6J mice

**Fig. 3:**
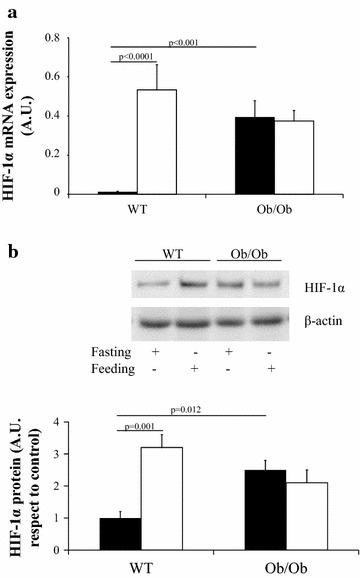
HIF-1α expression in visceral adipose tissue from wild-type C57BL6J (WT) and ob/ob C57BL6J mice (Ob/Ob) in fasting (*filled square*) (n = 17 in WT and n = 17 in Ob/Ob mice) and feeding status (*open square*) (n = 17 in WT and n = 17 in Ob/Ob mice). **a** HIF-1α mRNA and **b** representative immunoblot (n = 4 per group). The results are given as the mean ± SEM. *A.U.* arbitrary units

As humans are in a feeding status most of the day, we wanted to test whether this state produces a change in HIF-1α mRNA expression. We used C57BL6J wild-type mice and obese C57BL6J ob/ob mice, given the impossibility of accessing human VAT in a feeding status. In the fasting status, the behavior of VAT HIF-1α mRNA expression in C57BL6J mice was similar to human VAT. In fasting status, C57BL6J ob/ob mice had a higher HIF-1α mRNA expression than C57BL6J wild-type mice (p < 0.001) (Fig. 3a). In feeding status, C57BL6J wild-type mice had a similar HIF-1α mRNA expression than C57BL6J ob/ob mice (p = 0.091) (Fig. 3a). C57BL6J wild-type mice showed a significant increase in the expression of HIF-1α mRNA expression in feeding status respect to fasting status (p < 0.0001), and C57BL6J ob/ob mice did not show a significant change in the expression of HIF-1α mRNA expression in feeding status respect to fasting status (p = 0.535) (Fig. 3b). These results were confirmed by western blot analysis (n = 4 per group) (Fig. 3b).

## Discussion

This study shows that (a) VAT from morbidly obese subjects has a higher level of HIF-1α, (b) hypoxia alters the mRNA expression of genes involved in de novo lipogenesis in VAT, and (c) the postprandial status is associated with an increase in HIF-1α in VAT from C57BL6J wild-type mice.

HIF-1α, the major hypoxia-responsive gene, was increased in adipose tissue from morbidly obese subjects and obese mice, as in previous studies [5, 34]. This is in agreement with a study showing that adipose tissue is poorly oxygenated in obesity [35]. However, we do not have other techniques available in our laboratory to study VAT oxygenation, such as blood oxygen level-dependent magnetic resonance imaging, or staining with the chemical marker pimonidazole [10]. We also observed an association between human HIF-1α mRNA expression in human adipose tissue and HOMA-IR. In this respect, transgenic mice with constitutive activation of adipose HIF-1α developed insulin resistance and glucose intolerance [36]. In another study, hypoxia attenuated the insulin-stimulated glucose transport in mouse adipocytes [15]. Overall, our results suggest that VAT HIF-1α mRNA expression could be associated with the pathogenesis of insulin resistance.

A possible pathway that can be altered in the hypoxic condition is de novo lipogenesis. Reduced oxygenation of adipose tissue in obese humans has been associated with impaired insulin suppression of lipolysis [35]. PPAR-γ, the nuclear receptor involved in the regulation of lipid metabolism [37], is overexpressed in the adipose tissue of morbidly obese subjects [24]. Its association with hypoxia was confirmed in VAT explant cultures under hypoxic conditions, where increased PPAR-γ mRNA expression was found. However, opposite results were found with SREBP-1c [22, 38]. Our results in human VAT showed a decrease in SREBP-1c mRNA expression in morbidly obese subjects, in whom HIF-1α is increased. However, as in another study [38], increased SREBP-1c mRNA expression was found in VAT explant culture under hypoxic conditions. We do not know the reason for this discrepancy between the in vivo and in vitro results, but the n-6 polyunsaturated fatty acids could be involved, as they were increased in vivo in morbidly obese subjects [25] down-regulating the SREBP-1c expression [39, 40]. However, in the in vitro experiment, the fatty acid composition was similar in both normoxic and hypoxic conditions, and the effects produced by different fatty acids may be removed, with only the effects produced by hypoxia remaining.

Previous studies have shown that HIF-1α protein accumulation results in an energetic uncoupling via transcriptional repression of sirtuins [17]. We showed that SIRT3 mRNA expression is decreased in VAT from morbidly obese subjects and in the in vitro experiment in hypoxia. Low levels of SIRT3 could have important effects on the activity of other enzymes involved in de novo lipogenesis, since SIRT3 activates PDHB, ACSS2 and ACC1 by deacetylation [41].

The results obtained with VAT suggest a decrease in acetyl-CoA (a lower PDHB, ACSS2 and ACLY mRNA expression and enzymatic activity) and fatty acid synthesis (a lower ACC1 and FASN mRNA expression and malonyl-CoA concentration) in morbidly obese subjects. However, ACLY, ACC1 and FASN mRNA expression, ACLY activity and malonyl-CoA concentration was increased in the in vitro experiment in hypoxic conditions. We do not know the reason for the discrepancy between the in vivo and the in vitro results, but SREBP-1c could be involved. SREBP-1c is a transcription factor involved in the up-regulation of ACLY [42], ACC1 [43] and FASN mRNA levels [22, 40, 44]. SREBP-1c mRNA expression was decreased in VAT but increased in the in vitro experiment in hypoxic conditions, and this increase could perhaps be involved in the up-regulation of ACLY, ACC1 and FASN mRNA levels [22, 42, 43]. On the other hand, an increase in cytosolic citrate in hypoxia has been shown [45], and this could also increase the ACLY mRNA expression or activity, as we found. This would produce an increase in acetyl-CoA, the main precursor of fatty acid and mevalonate synthesis. Consequently, a high synthesis of acetyl-CoA from other enzymes, such as PDHB and ACCS2, would not be necessary. High levels of PDHB and ACSS2 should not be necessary, as we found. Although PDHB and ACSS2 mRNA expression is decreased in VAT from morbidly obese subjects, in whom HIF-1α is increased, 24 h hypoxia was not sufficient to significantly decrease the ACSS2 and PDHB mRNA expression or activity, as found in the in vitro experiment. However, the behavior of these enzymes in other lipogenic tissues may be different. A limitation of this study is that de novo lipogenesis was not directly measured by other techniques, such as ^3^H-water incorporation. Also, many of these enzymes are regulated by post-translational events.

Another important finding of this study is that the behaviour of obese mice was different to that of wild-type mice. As in morbidly obese subjects, VAT HIF-1α mRNA expression in obese mice was increased in the fasting status. In addition to hypoxia, this could be due to the more severe hyperinsulinemia of obese mice, since it is known that insulin increases HIF-1α mRNA in adipocytes [8], or other different stimuli [46]. This could also be the reason for the increased HIF–1α expression observed in the postprandial condition in wild-type mice. However, HIF-1α expression did not change in the postprandial condition in obese mice. This could be the same as in another study in which a similar blood flow was found before and after a meal in obese people [10]. However, there is little information on the effect of the postprandial state on VAT hypoxia. With these results, we could hypothesize that VAT from morbidly obese subjects would be in a hypoxic status all day (in fasting and in feeding status). This could have important repercussions on the pathways regulated by hypoxia in VAT, such as angiogenesis, cell proliferation, apoptosis, inflammation and insulin resistance [7, 8, 16, 17, 47, 48].

In conclusion, the results of the study showed that the VAT of morbidly obese subjects had an increased level of HIF-1α, a marker of hypoxia, which is negatively associated with the expression of genes involved in acetyl-CoA and fatty acid synthesis. Another important finding was that feeding status was associated with an increase in the VAT HIF-1α levels in C57BL6J wild-type mice, with a high level in both, fasting and feeding status in C57BL6J ob/ob mice. These persistently high levels of HIF-1α in obesity could have important repercussions on the pathways regulated by HIF-1α in VAT. Our results showed that VAT explants in the presence of hypoxia had a coordinated expression in genes involved in de novo lipogenesis pathways.

## Clinical perspectives

HIF-1α has been proposed to play a key role in the promotion and maintenance of dietary obesity. Most of these studies were conducted in animals and in other tissues different to VAT. However, there is very little reported on the influence of hypoxia on de novo lipogenesis in human VAT. The VAT of morbidly obese subjects had an increased level of HIF-1α, a marker of hypoxia. Another important finding was that the postprandial status in C57BL6J wild-type mice was associated with an increase in HIF-1α in VAT, with a high level in both, fasting and feeding status in C57BL6J ob/ob mice. In conclusion, hypoxia alters the mRNA expression of genes involved in de novo lipogenesis in VAT. These findings involve VAT in the metabolic consequences and comorbidities of obesity.
